# Machine learning to predict waitlist dropout among liver transplant candidates with hepatocellular carcinoma

**DOI:** 10.1002/cam4.4538

**Published:** 2022-01-14

**Authors:** Allison Kwong, Bilal Hameed, Shareef Syed, Ryan Ho, Hossein Mard, Sahar Arshad, Isaac Ho, Tashfeen Suleman, Francis Yao, Neil Mehta

**Affiliations:** ^1^ Division of Gastroenterology and Hepatology Stanford University Stanford USA; ^2^ Division of Gastroenterology University of California San Francisco California USA; ^3^ Division of Transplant Surgery University of California San Francisco California USA; ^4^ CloudMedx, Inc Palo Alto California USA

**Keywords:** liver cancer, liver transplant, outcome prediction, survival analysis, waitlist outcomes

## Abstract

**Background:**

Accurate prediction of outcome among liver transplant candidates with hepatocellular carcinoma (HCC) remains challenging. We developed a prediction model for waitlist dropout among liver transplant candidates with HCC.

**Methods:**

The study included 18,920 adult liver transplant candidates in the United States listed with a diagnosis of HCC, with data provided by the Organ Procurement and Transplantation Network. The primary outcomes were 3‐, 6‐, and 12‐month waitlist dropout, defined as removal from the liver transplant waitlist due to death or clinical deterioration.

**Results:**

Using 1,181 unique variables, the random forest model and Spearman's correlation analyses converged on 12 predictive features involving 5 variables, including AFP (maximum and average), largest tumor size (minimum, average, and most recent), bilirubin (minimum and average), INR (minimum and average), and ascites (maximum, average, and most recent). The final Cox proportional hazards model had a concordance statistic of 0.74 in the validation set. An online calculator was created for clinical use and can be found at: http://hcclivercalc.cloudmedxhealth.com/.

**Conclusion:**

In summary, a simple, interpretable 5‐variable model predicted 3‐, 6‐, and 12‐month waitlist dropout among patients with HCC. This prediction can be used to appropriately prioritize patients with HCC and their imminent need for transplant.

## Graphical abstract

Machine learning methods generated a simple, interpretable 5‐variable model to accurately predict 3‐, 6‐, and 12‐month waitlist dropout in patients with HCC. This prediction can be used to appropriately prioritize patients with HCC and their urgency for transplant.

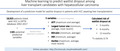

## 1 INTRODUCTION

Waitlist priority for patients with chronic liver disease is already based on urgency, using the Model for End‐Stage Liver Disease (MELD). However, most patients with hepatocellular carcinoma (HCC) have low predicted waitlist mortality based on MELD and access deceased donor liver transplantation (LT) by way of standardized exception points. Recognition of varying tumor behavior has spurred ongoing adjustments to the exception point system over the years, but all patients are still granted the same priority for LT regardless of tumor size or biology.^1^, ^2^, ^3^ It is increasingly clear that HCC tumor growth is heterogeneous, and that not all have the same risk of waitlist dropout or death––yet accurate risk stratification and prediction of waitlist outcome for these patients remain elusive.^4^, ^5^

Better risk stratification of waitlist dropout among LT candidates with HCC is needed in order to appropriately prioritize these patients. Both short‐ and long‐term outcomes in this population will be relevant to LT programs and help to guide strategic healthcare planning. In this study, we use machine learning methods to predict waitlist dropout among LT candidates with HCC in the United States.

## 2 METHODS

### 2.1 Data acquisition

All patients listed with a diagnosis of HCC in the United States in the standard OPTN STAR file were identified using primary diagnosis codes for HCC (4400 and 4401). Patients who received a deceased donor LT or were removed for death, clinical deterioration, or clinical improvement were included in the study. The latter group was included and considered as non‐events, as they often represent patients with HCC who respond to locoregional therapy and are at low risk of waitlist dropout. LT candidates listed with PELD were excluded. We included listings from March 1, 2002 up to December 31, 2017 to allow for adequate follow‐up.

Relevant clinical waitlist data including recipient characteristics and longitudinal laboratory values and imaging were extracted from the standard Organ Procurement and Transplantation Network (OPTN) Standard Transplant Analysis and Research files, which records all waitlist events and clinical updates for patients listed for transplantation in the United States. As part of the recertification to maintain HCC exception, LT candidates are mandated to have clinical updates every 3 months submitted to the OPTN, which include tumor size, number, and AFP. Specific types or timing of locoregional therapy were not considered, although changes in tumor size, number, and AFP over time served as a surrogate for response to therapy.

### 2.2 Data definitions and outcomes

The primary outcome was waitlist dropout at 3, 6, and 12 months after waitlist registration. Waitlist dropout was defined as patients who were removed from the transplant waitlist for death or clinical deterioration. Patients were censored at transplantation, and patients delisted for clinical improvement were censored at the time of removal from the waitlist.

Additional calculated features using longitudinal data included (1) total tumor area, defined as the sum of tumor area of all tumors in patient; (2) the size of largest tumor; and (3) the calculated Child–Turcotte–Pugh (CTP) score using the most recent INR, bilirubin, creatinine, ascites, and encephalopathy at each 3‐month timepoint.^6^ For patients with over five tumors, the calculation for total tumor area was limited to the five tumor sizes available in the OPTN database. For all continuous variables, the minimum, maximum, latest, and average were calculated at each 3‐month timepoint and entered as distinct variables. For categorical variables, binary features were created from the top six most common values. Including the calculated features, there were 1,181 variables available for analysis. Waitlist time was not considered as a predictor, since this would be directly influenced by external factors (e.g., changes in organ access during the study time period, as well as geographic variability) and thus mediate the outcome.

### 2.3 Data analysis

The primary analysis was a random forest model, a nonparametric tree‐based machine learning method that can account for interactions and non‐linearities in the data, using the Python package *scikit*‐*learn*. Values were normalized by standard deviation, and skewness was adjusted for in the outcome distribution by rebalancing. Missingness was imputed using median imputation. The random forest method for variable importance with 800 trees identified the top predictive variables, measured by mean decrease in accuracy and node impurity. Prediction accuracy was reported as the average area under the receiver operating curve (AUC) over 100 bootstrap samples, with 80% of the sample used for derivation and 20% for validation.

Spearman's rank correlation was performed on the subset of patients who were removed from the waitlist for death or clinical deterioration while awaiting LT. By considering only this group without access to transplant, this prediction represents the natural history of HCC without transplant and would theoretically be less affected by policy changes in liver allocation and prioritization and individual changes in access to LT during the study period.

In order to create a simple and clinically usable model, the features with the greatest predictive accuracy using these methods were selected for entry into the final model––a Cox proportional hazards model to predict the probability of waitlist dropout at 3, 6, and 12 months, using 20 randomly under‐sampled 80–20 train‐test splits. If patients in the derivation set had a transplant within 3, 6, or 12 months, they were censored at the time of transplant. Cox proportional hazards analysis is the method used to develop MELD for chronic liver disease and is particularly applicable in development of an urgency model intended to rank order patients based on their mortality risk without transplant.^7^ The classification performances of the model for dropout at 3, 6, and 12 months were ensembled over the 20 models and reported as the concordance statistic (c‐statistic). Accuracy was optimized by adding a second power of important features in the model to have higher weights during the modeling.

This study was performed in collaboration with CloudMedx, a health technology company focused on applications of machine learning methods in healthcare. As the data are already collected, deidentified, and available for research purposes from the OPTN (with an appropriate data use agreement), ethical approval was not sought from an institutional review board or ethics committee prior to commencing the study. All statistical analyses were performed using Python. A *p* value of <0.05 was considered significant, and all tests were two‐tailed.

## 3 RESULTS

There were 18,920 LT candidates listed with a diagnosis of HCC during the study time period who met inclusion criteria. In total, 3,476 patients (18.4%) were removed from the waitlist due to death or clinical deterioration while awaiting LT. The rate of dropout at 3, 6, and 12 months was 6.5%, 11.3%, and 17.2%, respectively. Cohort demographics are described in Table 1. The median age was 59 (IQR 54–64), 77.1% were male, the median biochemical MELD at listing was 12 (IQR 9–18), and median CTP score was 9 (IQR 8–10). The median follow‐up (waiting) time was 152 days (IQR 50–308).

**TABLE 1:** Cohort demographics (*n* = 18,920)

	*N* = 18,920
Age, years (IQR)	IQR 59 (54–64)
Sex, male (%)	77.1%
CTP score at listing (%)	
A	7851 (31.6%)
B	9439 (38.1%)
C	6385 (25.7%)
Missing	1139 (4.6%)
MELD at listing (IQR)	12 (9–18)
Serum bilirubin (mg/dL) (IQR)	1.6 (0.9–3.1)
Serum INR (IQR)	1.3 (1.1–1.6)
Total tumor diameter, cm (IQR)	4.66 (0.0–9.54)
Largest tumor size, cm (IQR)	2.8 (2.2–3.7)
Ascites (%)	
Absent	43.8%
Slight	38.8%
Moderate/large	12.8%
Missing	4.6%

In total, 1181 unique features derived from the OPTN data were considered in the random forest analysis. Both the random forest and Spearman's correlation analyses for 3‐, 6‐, and 12‐month waitlist dropout converged on 12 unique features comprised of 5 variables, which were selected for entry into the final model. These features included AFP as a continuous variable (maximum and average), largest tumor size (minimum, average, and most recent), bilirubin (minimum and average), INR (minimum and average), and ascites on a scale of 1–3 as reported to OPTN, 1 being absent, 2 being slight, and 3 being moderate/large (maximum, average, and most recent) (Table 2). MELD, CTP, and related component scores were noted to be strongly correlated with dropout; however, adding these to the model beyond the component variables that were already selected did not improve prediction accuracy and so were not included in the final model.

**TABLE 2:** Variables in final Cox regression model for waitlist dropout at 6 months

	Hazard ratio	95% CI
Alpha‐fetoprotein (ng/ml) (per 1 ng/ml increase)	1.00	1.00–1.00
Largest tumor size (cm), minimum	1.05	1.03–1.08
Largest tumor size (cm), average	1.02	1.01–1.03
Largest tumor size (cm), most recent	1.13	1.09–1.18
Serum bilirubin (mg/dl), minimum	1.05	1.02–1.09
Serum bilirubin (mg/dl), average	1.03	1.01–1.06
INR, minimum	0.59	0.49–0.71
Ascites, maximum	1.23	1.15–1.31
Ascites, average	0.85	0.80–0.91

The Cox proportional hazards model predicted the risk of dropout at 3, 6, and 12 months in the validation set with a c‐statistic of 0.74. To illustrate the differential risk, the risk of waitlist dropout for a patient with compensated liver disease (INR 1.0, bilirubin 1.0 mg/dl, no ascites), AFP 100 ng/ml, and a single 3.0 cm tumor would have a low calculated dropout risk of 4% at 3 months, 8% at 3 months, and 18% at 12 months. After demonstrated response to locoregional therapy with AFP of 5 ng/mL and no viable tumor on the next scan, this patient would then have a calculated dropout risk of 3% at 3 months, 7% at 6 months, and 15% at 12 months (Table 3). In contrast, a patient with decompensated cirrhosis (INR 2.0, bilirubin 3 mg/dL, moderate ascites), AFP of 400 ng/mL, and 3 cm of viable tumor would have a calculated dropout risk of 17% at 3 months, 32% at 6 months, and 59% at 12 months. An online calculator based on this model was created for clinical use and can be found at: http://hcclivercalc.cloudmedxhealth.com/.

**TABLE 3:** Specific combinations of input variables and the calculated risk of dropout

		Patient 1^a^	Patient 2^b^	Patient 3^c^	Patient 4^d^	Patient 5^e^
Alpha‐fetoprotein (ng/ml), maximum		400	100	100	800	400
Alpha‐fetoprotein (ng/ml), average		400	100	55	400	85
Largest tumor size (cm), minimum		3.0	3.0	10	3.0	0
Largest tumor size (cm), average		3.0	3.0	1.5	3.0	4.0
Largest tumor size (cm), most recent		3.0	3.0	0	3.0	0
Serum bilirubin (mg/dl), minimum		3.0	1.0	1.0	1.0	1.0
Serum bilirubin (mg/dl), average		3.0	1.0	1.0	1.0	2.0
INR, minimum		2.0	1.0	1.0	1.0	1.0
INR, average		2.0	1.0	1.0	1.0	1.5
Ascites, maximum		Moderate	None	None	None	Slight
Ascites, average		Moderate	None	None	None	Slight
Ascites, most recent		Moderate	None	None	None	Slight
Predicted probability of dropout	3 months	17%	4%	3%	5%	10%
	6 months	32%	8%	7%	10%	19%
	12 months	59%	18%	15%	21%	39%

^a^ Decompensated liver disease, high AFP with 3 cm viable tumor.

^b^ Compensated liver disease, single, 3 cm lesion.

^c^ Compensated liver disease, single, 3 cm lesion, with response to locoregional therapy.

^d^ Compensated liver disease, high AFP with 3 cm viable tumor.

^e^ Decompensated liver disease, downstaged HCC with AFP response.

## 4 DISCUSSION

Better risk stratification can identify those patients with HCC with greater urgency for LT. Our parsimonious 5‐variable model predicted 3‐, 6‐, and 12‐month waitlist dropout among patients with HCC with a c‐statistic of 0.74. The analysis included all available variables directly collected in a large national database of all LT waitlist events since 2002, as well as a number of calculated features including average, minimum, and maximum, for a total of 1181 features. We evaluated dynamic longitudinal data involving laboratory values and imaging characteristics, so that clinical information could be entered at any timepoint during the waitlist period but also still consider initial tumor characteristics and response to locoregional therapy. The resulting five variables in the final model, including AFP, tumor size, bilirubin, INR, and ascites, represent the most predictive risk factors in this population. These variables are clinically relevant, representing both the severity of liver disease and the burden of HCC.^8^, ^9^ Patients with these risk factors are at higher risk of waitlist dropout due to the combined risk of liver failure and/or progression of HCC, with limited options for locoregional therapy due to the risk of hepatic decompensation while awaiting LT.

Together, these variables and their trajectory over time were combined to generate a predictive model to estimate the risk of waitlist dropout at 3‐, 6‐, and 12‐month time horizons. The c‐statistic of 0.74 is comparable to other proposed models, including a recently proposed waitlist dropout score by Mehta et al. based on listing variables (0.74 for LWR, 0.71 for MWR, and 0.73 for SWR).^10^ This model can help to risk stratify those patients with HCC with greater urgency for LT, versus those with more indolent disease who may be able to wait. We propose that a 6‐month probability of waitlist dropout of ≤10% would be considered low risk, 10%–15% moderate risk, and ≥15% high risk––wherein a high‐risk patient would derive greater benefit from timely LT, whereas a low‐risk patient less so. These proposed values reflect the observed range of waitlist dropout risk at 6 months in the development cohort of the abovementioned risk score, which ranged from 3.6% in the lowest risk quartile and up to 28.1% in the highest risk quartile, with an overall dropout risk of 10.8% at 6 months. These thresholds may vary depending on local resources and donor availability, which can be influenced by recipient size, blood type, and geography.

Donor livers are a scarce resource in the United States. This predictive model can help to better risk stratify LT candidates with HCC and define their urgency for LT. Under the current US system, which was implemented in 2018, all local patients are assigned a static score (median MELD at transplant minus 3)––and so all LT candidates with HCC are considered equal. The policy in place prior to this––the “MELD escalator”––was also a fixed scoring system, which increased uniformly based on waitlist time rather than tumor burden or characteristics. However, there is clearly a differential risk of waitlist dropout and urgency based on dynamic patient and tumor characteristics, and those with a higher risk of waitlist dropout could be granted higher allocation priority. Such a system has been proposed and implemented in Québec, Canada, with more exception points granted to those with increased number and/or size of tumor, and no observed adverse effect on graft or patient survival.^11^ In the context of organ shortage, living donor LT and expanded criteria donor options may also be appropriate options to explore for those identified to have a higher dropout risk and thus greater urgency for LT. Extended criteria, or “marginal” livers, for example, donation after circulatory death or steatotic liver grafts, are potentially associated with inferior posttransplant outcome, but may still confer an overall survival benefit particularly for patients with HCC in greatest need of transplant––who still generally have lower waitlist priority relative to patients with chronic liver disease in the current US allocation system.^12^, ^13^

It must be noted that prioritization of those at higher risk of waitlist dropout will need to be balanced against the individual risk of post‐LT HCC recurrence. In a recent study considering the effect of listing characteristics including tumor size and number, AFP, CTP, and MELD‐Na score on post‐LT survival, inferior 5‐year post‐LT survival was observed in the highest risk stratum.^10^ However, below this threshold, the risk of waitlist dropout could be stratified without significant differences in post‐LT survival. In contrast, our model leverages dynamic waitlist data––representing the evolution of liver disease and response to therapy during the waiting period––to evaluate dropout risk in real time and can be used in conjunction with existing scores such as this to estimate the overall survival benefit.

Traditional linear or survival methods that have been previously applied to this dataset are more limited in scale, require parametric specification, and may not recognize high‐order interactions. The random forest method can handle a large number of variables and account for complex interactions and non‐linearities in the underlying data. A previous Cox proportional hazards model for waitlist dropout using OPTN data identified MELD, maximum tumor size, and AFP as important predictors of waitlist dropout at 3 months, with a c‐statistic of 0.78.^14^ Duvoux et al. proposed the French AFP model, comprised of AFP, tumor size, and number, to predict HCC recurrence, with an AUC of 0.70.^15^ A more recent model using competing risk analysis, with an endpoint of waitlist dropout at 3 months, added in age, number of tumors, and etiology of liver disease, resulting in a c‐statistic of 0.72.^16^ Prediction models to stratify longer term dropout risk have not been widely investigated.

Prediction models that use machine learning methods have gained traction in recent years.^17^ Using a similar tree‐based method and a total of 28 variables, Bertsimas et al. demonstrated superior performance of their OPOM model compared to MELD‐Na for the prediction of 3‐month waitlist mortality.^18^ In simulation, this model allocated more livers to non‐HCC waitlist candidates and improved waitlist outcomes for both HCC and non‐HCC patients, compared to MELD‐Na. Our proposed model adds to this literature and is targeted for patients with HCC, incorporating tumor‐specific variables and a longer time horizon. The model also accounts for dynamic longitudinal data, so that clinical information could be entered from just one timepoint (i.e., listing) but also additional timepoints before or after. Data could be entered during the waiting period, with the relevant longitudinal values (e.g., minimum, maximum, and average) being calculated over all previous timepoints provided, and the prediction would be made for 3‐, 6‐, and 12‐month survival from the current time. This type of risk stratification may help to inform center‐level clinical decision‐making regarding waitlist management.

Limitations of this study include its retrospective nature and reliance on the UNOS database, which is relatively comprehensive but potentially susceptible to data entry errors and inconsistencies. Uncaptured variables such as PIVKA‐II, degree of differentiation, and type of locoregional therapy may also influence the natural history and outcomes of HCC but are not considered in OPTN‐derived models. These variables are not readily available for most liver transplant candidates with HCC in the United States and so are not yet practical for use in clinical prediction modeling. Response to locoregional therapy was indirectly represented by tumor size and number over time. Although there is preliminary data to suggest that outcomes may be worse in patients with HCC and liver disease due to nonalcoholic steatohepatitis or hepatitis C virus, the etiology of liver disease was not predictive in our analysis and so was not included in the prediction model.^19^, ^20^ Center variation in thresholds and risk tolerance for bridging locoregional therapy, as well as changes in organ allocation and access to transplant, could also influence the predicted waitlist outcome. In addition, patients by design contributed varying amounts of data based on the length of waitlist time, which could have introduced some bias to the model development. The median waiting time in this cohort was 152 days (IQR 50–308), meaning that most patients contributed data from more than one timepoint. Consequently, this model may be more representative of outcomes in regions with longer wait times, whose patients had the opportunity to contribute more waitlist data. Waiting times for LT candidates with HCC have lengthened in recent years, and the majority are now subject to a mandated waiting period of at least 6 months prior to LT, making this aspect of the model relevant to the current allocation system. Much of the study period predated widespread adoption of a standardized downstaging protocol in the United States, and so outcome prediction of larger tumors may be less stable. Finally, while the model was internally validated, external validation is necessary to establish its generalizability, particularly outside of the United States with differing allocation policies for patients with HCC.

In summary, this 5‐variable model effectively predicts 3‐, 6‐, and 12‐month waitlist dropout among patients with HCC. These variables identified as the strongest predictors represent both the severity of underlying liver disease and tumor burden. Models developed using machine learning methods can identify important predictors and help to guide center‐level clinical decision‐making and organ acceptance practices. This calculator can be applied to risk stratify LT candidates with HCC and recognize the patients who are at highest risk of waitlist dropout while awaiting LT.

## 5 ETHICS STATEMENT

As the data are already collected, deidentified, and available for research purposes from the OPTN (with an appropriate data use agreement), ethical approval was not sought from an institutional review board or ethics committee for this study.

## CONFLICT OF INTEREST

The authors have no conflict of interest to declare.

## DISCLOSURE

This work was supported in part by Health Resources and Services Administration contract HHSH250‐2019‐00001C. The content is the responsibility of the authors alone and does not necessarily reflect the views or policies of the Department of Health and Human Services, nor does mention of trade names, commercial products, or organizations imply endorsement by the U.S. Government.

## AUTHOR CONTRIBUTIONS

AJK: Study concept and design; interpretation of data; drafting of manuscript. BH: Interpretation of data; critical revision of the manuscript for important intellectual content. SS: Interpretation of data; critical revision of the manuscript for important intellectual content. RH: Analysis of data, participated in performance of the research. SA: Analysis of data, participated in performance of the research. TS: Analysis of data, participated in performance of the research. FY: Interpretation of data; critical revision of the manuscript for important intellectual content. NM: Study concept and design; analysis and interpretation of data; drafting of manuscript; critical revision of the manuscript for important intellectual content; study supervision.

## Data Availability

The data that support the findings of this study are available from the Organ Procurement and Transplantation Network. Restrictions apply to the availability of these data, which were used under license for this study, and so are not publicly available.
